# Identifying hydro-meteorological events from precipitation extremes indices and other sources over northern Namibia, Cuvelai Basin

**DOI:** 10.4102/jamba.v7i1.177

**Published:** 2015-10-30

**Authors:** Frans C. Persendt, Christopher Gomez, Peyman Zawar-Reza

**Affiliations:** 1 Department of Geography, University of Canterbury, New Zealand; 2 Department of Geography, History and Environmental Studies, University of Namibia, Namibia

## Abstract

Worldwide, more than 40% of all natural hazards and about half of all deaths are the result of flood disasters. In northern Namibia flood disasters have increased dramatically over the past half-century, along with associated economic losses and fatalities. There is a growing concern to identify these extreme precipitation events that result in many hydro-meteorological disasters. This study presents an up to date and broad analysis of the trends of hydro-meteorological events using extreme daily precipitation indices, daily precipitation data from the Grootfontein rainfall station (1917–present), regionally averaged climatologies from the gauged gridded Climate Research Unit (CRU) product, archived disasters by global disaster databases, published disaster events in literature as well as events listed by Mendelsohn, Jarvis and Robertson (2013) for the data-sparse Cuvelai river basin (CRB). The listed events that have many missing data gaps were used to reference and validate results obtained from other sources in this study. A suite of ten climate change extreme precipitation indices derived from daily precipitation data (Grootfontein rainfall station), were calculated and analysed. The results in this study highlighted years that had major hydro-meteorological events during periods where no data are available. Furthermore, the results underlined decrease in both the annual precipitation as well as the annual total wet days of precipitation, whilst it found increases in the longest annual dry spell indicating more extreme dry seasons. These findings can help to improve flood risk management policies by providing timely information on historic hydro-meteorological hazard events that are essential for early warning and forecasting.

## Introduction

**FIGURE 1:**
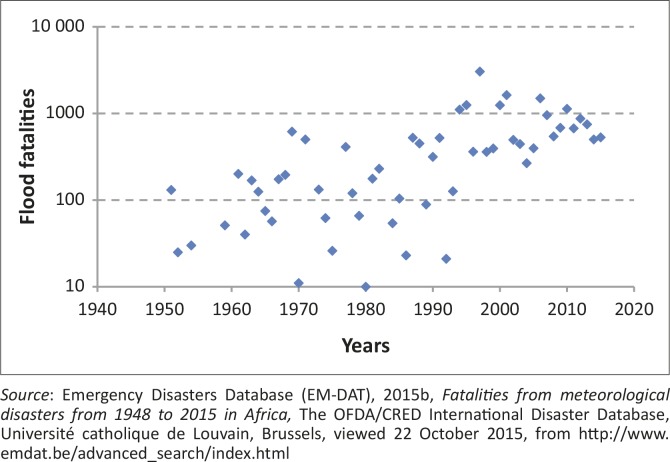
Statistics of fatalities by flood disaster events from 1950 to 2015 in Africa.

More than 40% of all natural hazards and about half of all deaths worldwide are the result of flood disasters (Emergency Disasters Database [EM-DAT] 2015a; Ohl & Tapsell 2000). In recent decades, floods have caused much damage economically and have also affected millions of people, especially in developing countries (Jonkman 2005; Jonkman, Vrijling & Vrouwenvelder 2008). Of the disaster-related fatalities 31% were caused by floods worldwide, between 1900 and 2006, and these fatalities are second only to drought (53%) (Smith 2013). In Africa, research has shown that between 1950 and 2010, floods caused an increase in fatalities by one order of magnitude (Figure 1; Di Baldassarre *et al*. 2010; EM-DAT 2015b). But studies have also found no evidence that the magnitude of African floods has increased during the 20th century (Di Baldassaree *et al*. 2010). Yet, others have projected an expected increase in the risk of floods in tropical Africa (Hirabayashi *et al.* 2008).

**FIGURE 2:**
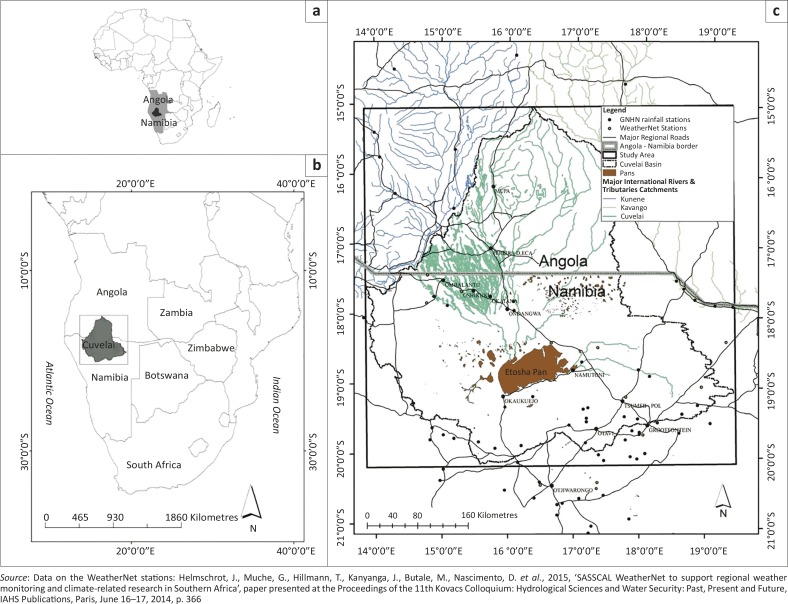
(a) Location of the Cuvelai River Basin in Africa. (b) The location of the CRB in southern Africa and (c) the CRB with the location of the study area (box) between southern Angola and northern Namibia. The figure also shows all the national rainfall stations as well as the new automatic rainfall stations from the WeatherNet.

**FIGURE 3:**
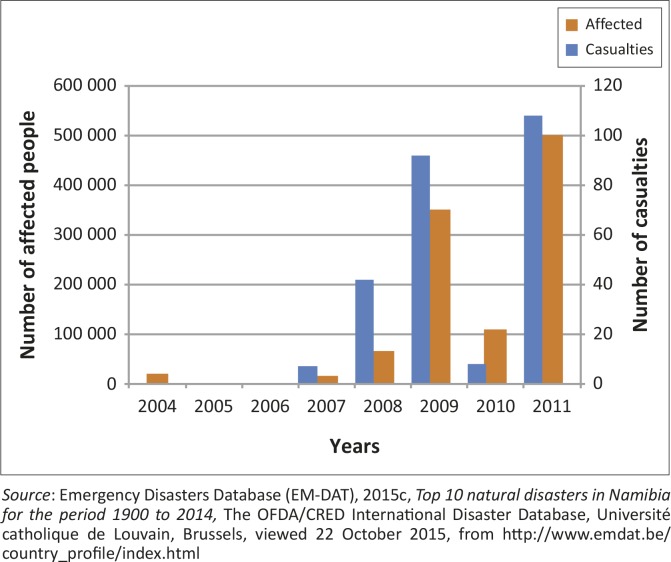
Inventory data for Namibia from 1900–2014 showing the number of people affected and the casualties caused by flooding.

In northern Namibia (Figure 2), flood disasters have increased dramatically over the past years, along with associated economic losses, and fatalities (Figure 3; EM-DAT 2015c; Kundzewicz *et al*. 2014). Many causes for flooding are possible hence most flood disaster events differ widely depending – for example – on the climate and the geography of the drainage basin, amongst other causes (Hofer & Messerli 2006; Smith 2013).

There is a low to medium confidence in the historical extreme rainfall trends observed over most of Africa, because of partial lack of data, lack of literature, and inconsistency of reported patterns in the literature (Conway *et al.* 2009; Seneviratne *et al*. 2012). Observed changes (from ground-based rainfall stations) have been reported for areas extending from Namibia to Angola: including a reduction in late austral summer precipitation as well as an increasing trend in daily rainfall intensity, which has implications for run-off characteristics (Hoerling *et al.* 2006; New *et al*. 2006). Extreme precipitation linked to climate change is one of the most widely accepted causes of flood events (Abbas *et al.* 2014; Simonovic 2012; Teegavarapu 2012), as a result of this the World Meteorological Organisation/Climate Variability and Predictability (WMO/CLIVAR) Expert Team on Climate Change Detection, Monitoring and Indices (ETCCDMI 2013) has conducted initial studies on worldwide analysis of both observed temperature and precipitation daily extreme indices. These studies showed noteworthy observed changes in extreme climate indices, for example, they found – during the last 50 years – major increases in heavy rainfall events (Alexander & Arblaster 2009; Alexander *et al.* 2006; Frich *et al.* 2002). Whilst research from southern Africa has shown more variable patterns for all extreme daily precipitation indices except for the maximum 5–day rainfall that presented a consistent pattern of increase, especially during the second half of the twentieth century (Frich *et al.* 2002).

More research has been undertaken on the observed monthly climate over the southern Africa region resulting from the scarcity and paucity of daily precipitation data. Unganai and Mason (2001) have provided a regional and national overview on recent trends and variability in the monthly climate over Africa. New *et al.* (2006) have provided the first regional synthesis of trends in daily extremes for southern Africa using extreme precipitation daily indices and they have also provided a very good regional synthesis of extreme precipitation trends over Namibia. In South Africa, many studies on extreme precipitation exist, amongst these Kruger (2006) focused on 138 South African rainfall stations using the indices described in New *et al.* (2006), whilst Mason *et al.* (1999), investigated trends in extreme precipitation that had not experienced locational changes – but without testing for other inhomogeneities. They identified significant increases in the intensity of extreme rainfall events between 1931–1960 and 1961–1990 over 70% of South Africa.

Future projections for southern Africa are:

1. medium confidence – that the projected droughts will intensify in the 21st century in some seasons, resulting from reduced precipitation or increased evapotranspiration
2. low confidence – that the projected heavy precipitation will increase. The projected changes for the climatological dry southwest parts of southern Africa have signalled drying in the annual mean as well as during the austral summer months (James & Washington 2013; Moise & Hudson 2008; Orlowsky & Seneviratne 2012).

Furthermore, a projected delay in the onset of seasonal rains in southern Africa is caused by rainfall decreases during austral spring months, and this is also projected for the region (Seth *et al.* 2011; Shongwe *et al*. 2009).

**FIGURE 4:**
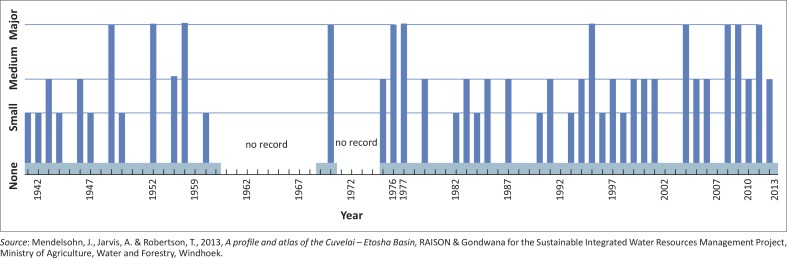
Flood levels from year-to-year in the CRB, northern Namibia.

In countries qualified as ‘developing’, such as Namibia, observed climate change time series data and detailed quantitative flood events archives are usually unavailable (Filali-Meknassi, Ouarda & Wilcox 2014). Qualitative data have been used to derive historical flood events from 1941 to 2013 (Figure 4). Different sources were used to compile this archive (Figure 4) such as flow and flooding extent (levels) as well as fish species types and abundance within the *Oshanas* (channels) of the CRB (Mendelsohn *et al.* 2013). The former were derived from hydrography and observational studies that recorded the occurrences of flooding between 1941–1942 and 1960–1961 within the CRB (Stengel 1963) whilst the latter were derived from fish species’ type and abundance studies that were used to categorise the magnitude of the flood events between 1975–1976 and 1989–1990 (Van der Waal 1991). Even though this is the most up to date historical record of flood events for the CRB, for the last 73 years, numerous data gaps remain present, for example, no data are available for 13 years. Flood events, in this archive, are qualitatively classified into three classes:

- exceptionally high flows (major flood events) for 11 years: 1949, 1953, 1956, 1970, 1976, 1977, 1995, 2004, 2008, 2009 and 2011
- 18 years of medium flows (medium floods)
- whilst no or only negligible flow (small floods) for 12 years were reported (Figure 4).

This archive has also been used to better understand the flooding occurrences in the CRB (Hipondoka 2005; Mendelsohn, el-Obeid & Roberts 2000; Mendelsohn *et al.* 2009; Miller, Pickford & Senut 2010), however, for many years no record of hydro-meteorological disasters have been reported. This study validates and appends possible disaster events for the period 1941–2013, using data from various sources especially for the period from 1960 to 1968 that has no records.

Extreme precipitation events play an important role in monitoring and predicting the occurrence of flood disaster events (Hofer & Messerli 2006; Wuensch & Curtis 2010). Furthermore, the evaluation of short and long duration precipitation events is also critical to our understanding of flooding and its impact on natural and built environments (Teegavarapu 2012). Also heavy precipitation (pluvial flooding) in agriculture delays spring planting, increases soil compaction and causes crop losses through anoxia and root diseases, thus, identifying these heavy precipitation events is essential. Additionally, linking rain-induced flooding to certain extreme precipitation events will help with adaptation and improve coping mechanisms. This is especially significant in the low-relief landscape such as the Cuvelai basin that has many pans, which compound the severity of pluvial flooding, as heavy downpours cause these pans to overflow and inter-link, causing flooding in the absence of vegetation which buffers run-off (Jury & Engert 1999).

Hence, the aim of this article is to develop an up to date archive of flood events by comparing referenced flood events listed above to events (years) derived from:

- extreme precipitation indices calculated from data obtained from the Grootfontein rainfall station (longest continuous time series data for northern Namibia)
- regional rainfall stations (New *et al.* 2006)
- global disasters databases
- rainfall climatologies of the Grootfontein station
- averaged data (area and time – annual) from the CRU TS 3.21 product (Harris *et al.* 2014; Mitchell & Jones 2005).

This article contributes towards supplementing geospatial data scarcity that delays the implementation of an efficient and effective flood risk management system in Namibia and also the advancement of climate change adaptation strategies. An updated flood disaster event archive is also essential as flood disaster events are short lived and their spectacular impact is soon forgotten, thus, awareness of the flood disaster event quickly subsides (Barnolas & Llasat 2007).

## Study area

**FIGURE 5:**
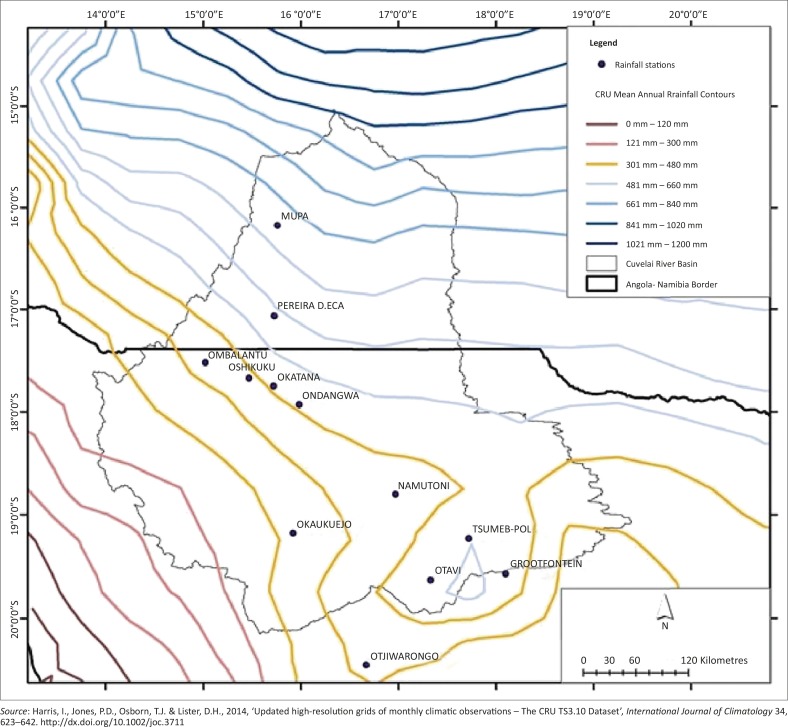
Mean annual rainfall for the CRB derived from CRU 3.21 data. The original data covered a period from 1901–2012 at a gridded spatial resolution of 0.5° and were converted into contours.

The CRB is an area of 167 600 km^2^ and is located between 14°E and 15° E longitude, and 15°S and 20° S latitude. It extends over 450 km from north to south and is located between Angola and Namibia. It is surrounded by the Kunene and Okavango rivers to the north-west and north-east respectively (Figure 2). The administrative regions of the basin are comprised of the Omusati, Ohangwena, Oshikoto and Oshana areas in Namibia and the Kunene region in Angola. The basin has a semi-arid climate in the southern part (Namibia) and a subtropical climate in the northern part (Angola), with an annual rainfall ranging from less than 200 mm – 900 mm (Figure 5; Mendelsohn & Weber 2011). The rainfall is highly variable and falls in the austral summer (October – April) mainly resulting from the seasonal north-south movement of the Inter-Tropical Convergence Zone (ITCZ) that brings moist tropical air southwards. During this time, maximum daily temperatures can range between 35 °C – 40 °C whilst evaporation and evapotranspiration rates are also at the annual highest.

The dominant vegetation types of the area are the *Colophospermum* mopane shrubland and woodland (northern part), whilst the saline grasslands are found within different landscapes of the southern part (Kangombe 2010). These landscapes include: the Cuvelai, mopane shrubland, Karstveld, eastern and west Kalahari woodlands, salt pans and surrounding plains (Mendelsohn & Weber 2011; Mendelsohn *et al.* 2009). The study area is densely populated, having the highest population density in Namibia of approximately 100 people per km^2^, and more than 50% of the population of Namibia lives in this area (Mendelsohn & Weber 2011; Mendelsohn *et al.* 2000; Mendelsohn *et al.* 2009). It is also host to Namibia's fastest growing rural areas where livelihood strategies for the vast majority of people are from the land (Mendelsohn & Weber 2011). The land-uses in this region are: rain-fed subsistence farming that is vulnerable to recurring droughts and floods; firewood for energy and building purposes and water for livestock and human consumption. In the southern part, surface water storage facilities are limited resulting from the low topography, high evaporation rates and shallow salty groundwater. The soils in the study area are: sandy, porous and infertile. Hence, these soils have a low probability for use in crop farming but are good for rangeland farming (Mendelsohn *et al.* 2009), whilst an impermeable layer below the sand compounds the problem (Mendelsohn & Weber 2011). Furthermore, where this layer occurs, vast areas are inundated with water when it rains for long periods. Additionally, some areas (southern part) have multiple broad channels as well as numerous pans that are composed of waterborne clay which produced fertile soils (cambisols and calcisols) for many agricultural uses (Mendelsohn & Weber 2011). Lastly, the Mupa National Park (Angola) and the world-renowned Etosha National Park (Namibia) are located within the basin.

## Objectives

A sound and reliable archive on floods and flooding is important to insurance companies, research institutes, and government and financial organisations that can benefit from accurate data. It is also useful for more appropriate and suitable flood management and protection measures, both structural and non-structural, for analysing locational strategies for disaster risk mitigation. Furthermore, it will help institutions optimise their investments to alleviate poverty and to stimulate (economic) prosperity and, finally, many stakeholders are also interested in collecting correct information to improve their knowledge on floods and flooding.

The specific objectives of this article are to:

1. list flood events according to their magnitudes (Table 1 & Figure 4)
2. document flood events published in literature based on hydro-meteorological data
3. calculate extreme precipitation indices for the Grootfontein station, and compare them to the regional trends (New *et al.* 2006)
4. calculate climatologies for the Grootfontein rainfall station
5. calculate area averaged climatologies and also anomalies for the CRB using the CRU product data
6. validate the listed flood events (Table 1) against those identified from other sources.

**TABLE 1:** Reported flood events in the Cuvelai basin for the last 73 years from 1941 to 2013.

Magnitude of flood disaster	Years
High flows occurred in 11 years (major flood disaster events)	1949, 1953, 1956, 1970, 1976, 1977, 1995, 2004, 2008, 2009, and 2011
Medium flows occurred in 18 years (medium floods)	1943, 1946, 1955, 1975, 1979, 1983, 1985, 1987, 1991, 1994, 1997, 1999, 2000, 2001, 2005, 2006, 2010, and 2012
Negligible flow occurred in 12 years (small floods)	1941, 1942, 1944, 1947, 1950, 1958, 1982, 1984, 1990, 1993, 1996, and 1998
No flow occurred in 18 years	1945, 1948, 1951, 1952, 1954, 1957, 1959, 1969, 1978, 1980, 1981, 1986, 1988, 1989, 1992, 2002, 2003 and 2013
No data available in 13 years	1960, 1961, 1962, 1963, 1964, 1965, 1966, 1967, 1968, 1971, 1972, 1973, and 1974

*Source*: Mendelsohn, J., Jarvis, A. & Robertson, T., 2013, *A profile and atlas of the Cuvelai – Etosha Basin,* RAISON & Gondwana for the Sustainable Integrated Water Resources Management Project, Ministry of Agriculture, Water and Forestry, Windhoek

## Data and methods

### Data

This article has used, as reference, an existing list of flood events (Table 1) to compile an up to date archive of past flood events. It has also used the referenced events for comparison to events obtained from other sources.

The sources used in this article were:

1. flood events from the archive (Figure 4) and reproduced in Table 1
2. daily rainfall data from the Grootfontein station that will be used to calculate these indices as utilised by New *et al.* (2006)
3. daily rainfall data to calculate climatologies (Grootfontein)
4. information from the global disaster databases such as the EM-DAT
5. rain-gauged gridded precipitation (CRU) data
6. published literature on flood events in northern Namibia.

Table 1 summarises the known flood events for the last 73 years from 1941 to 2013. For this period the following flood events were reported:

- exceptional high flows (major floods events) – 11 times
- medium flows (medium floods) – 18 times
- negligible flow (small floods) – 12 times
- no flow – 18 times
- years with no data – 13 times.

These flood events (years) were used as references to validate results obtained from other sources that enabled the creation of an up to date archive of flood events for the study area. The archive of hydro-meteorological events (droughts and floods) will help to identify weather and climate extreme events that have caused large flood disasters in the study area. This is especially relevant as a changing and variable climate can lead to changes in the frequency, intensity, spatial extent, duration, and timing of these events (Niang *et al.* 2014).

Rainfall daily data from several stations in the Cuvelai basin were evaluated using two criteria. Firstly, the selection of rainfall gauge stations must have at least 30 years of data over a long-term period, including the climate (normal) standard period from 1961 to 1990. Secondly, the stations must have limited missing data (less than 10%) in the overall long-term record (New *et al.* 2006). The Grootfontein rainfall station is the only station that meets these criteria in northern Namibia. The data for the Grootfontein station were obtained from the daily Global Historical Climatology Network (GHCN-Daily) data set (Alexander *et al.* 2006; Gleason *et al.* 2002) as well as from the Namibia Meteorology Service (NMS 2015b). After selection, the data of the Grootfontein rainfall station were checked for erroneous data using data plots that enabled visual inspection. Local meteorological knowledge proved crucial in assessing a number of large precipitation outliers (compared to surrounding rainfall stations), whilst data plots were also used to detect larger temporal inhomogeneities (Klein-Tank, Zwiers & Zhang 2009; Kruger 2006; New *et al.* 2006). The Grootfontein data were used to calculate the climate change extreme indices as well as annual climatologies.

The Emergency Disasters Database (EM-DAT 2015a) is the most commonly used global database and was used to obtain flood events for the CRB (Adhikari *et al.* 2010; Smith 2013; Tschoegl, Below & Guha-Sapir 2006). For the period 1900 to 2014, the EM-DAT (2015c) lists only four flood disaster events for the top ten natural disasters in Namibia: 2008, 2009, 2010 and 2011 (Figure 3).

Annual precipitation climatologies and composites from the CRU product (Harris *et al.* 2014; Mitchell & Jones 2005) were calculated. This monthly gridded product covers the period 1901–2012 and is composited of observations at meteorological stations across the world's land areas. The spatial resolution of the product is 0.5° longitude by 0.5° latitude. The dataset was used because it represents the longest temporally consistent global dataset that covers the CRB. Lastly, published literature of reported flood events in the Cuvelai basin was also compiled.

### Methods

Flood events were identified from climate change extreme precipitation indices. Extreme precipitation indices are valuable for climate monitoring but are not recommended for use for activities such as weather forecasting (Klein-Tank *et al.* 2009). The RClimDex package (Expert Team on Climate Change Detection Monitoring and Indices [ETCCDMI 2013]) was used to perform quality control on the daily Grootfontein rainfall station data. The duration, frequency, extent, intensity and total amount of extreme long-term rainfall are crucial in identifying past and predicting future hydro-meteorological hazard events. This study used this package to calculate ten standardised indices (annually) that have proven to assess and analyse these characteristics of rainfall in order to understand and monitor a changing climate. Additionally, these indices are also designed to allow for the regional comparison of these characteristics (Klein-Tank *et al.* 2009). The indices are different from the traditional measures of extremes, in statistics, in that they record the occurrences of several extreme events per year whilst measures of extremes capture only the extreme value in a distribution's tail, hence they exclude some years (Alexander *et al.* 2006; New *et al.* 2006). The indices used for the present contribution can be classified into four categories:

1. precipitation frequency (R20 mm and R10 mm)
2. precipitation intensity (RX1day, RX5day, simple daily intensity index [SDII], R99p and R95p)
3. precipitation duration (consecutive dry days [CDD] and consecutive wet days [CWD])
4. precipitation amount (PRCPTOT).

Alternatively, the indices can be classified by their:

- percentile (R95p and R99p)
- absolute (RX1day, RX5day, R10 mm and R20 mm)
- duration (CDD and CWD) values even though the total cumulative precipitation (PRCPTOT) and SDII do not fit this classification.

Percentile-based indices (very wet days – R95p and extremely wet days – R99p) represent the amount of rainfall falling above the 95th (R95p) and 99th (R99p) percentiles. They also include, but are not limited to, the most extreme precipitation events in a year. Absolute indices represent maximum or minimum values within a year and they include the maximum 1–day precipitation amount (RX1day) and maximum 5–day precipitation amount (RX5day). Threshold indices, in contrast, are defined as the number of days on which a precipitation value falls above or below a fixed threshold and include the annual occurrence of the number of heavy precipitation days greater than (>) 10 mm (R10 mm) and the number of very heavy precipitation days > 20 mm (R20 mm). Duration indices are defined as periods of excessive wetness or dryness and they include the indices of the CDD and the CWD. The CDD index is defined as the length of the longest dry spell in a year whilst the CWD index is the longest wet spell in a year. Other indices include annual precipitation total (PRCPTOT) and the SDII. They do not fall into any of the above categories but changes in them could have significant societal impacts (Klein-Tank *et al*. 2009).

Values for the ten indices were calculated from the Grootfontein daily rainfall station data. Also, linear trends for these indices for both the Grootfontein station as well as the regional rainfall stations (New *et al.* 2006) are listed in Table 2.

**TABLE 2:** The ten climate change extreme daily indices used for this study.

Index	Description	Definition	Units	Grootfontein Station Trend	Regional Trend
PRCPTOT	wet day precipitation	annual total precipitation from wet days	mm	Positive	Negative
SDII	simple daily intensity index	average precipitation on wet days	mm/d	Negative	Positive
CDD	consecutive dry days	maximum number of consecutive dry days	days	Positive	Positive
CWD	consecutive wet days	maximum number of consecutive wet days	days	Positive	Negative
R10 mm	heavy precipitation days	annual count of days when RR >= 10	days	Negative	Negative
R20 mm	very heavy precipitation days	annual count of days when RR >= 20	days	Positive	Negative
R95p	very wet day precipitation	annual total precipitation when RR > 95th percentile of 1961–1990 daily rainfall	mm	Negative	Positive
R99p	extremely wet day precipitation	annual total precipitation when RR > 99th percentile of 1961–1990 daily rainfall	mm	Positive	Positive
RX1day	maximum 1–day precipitation	annual maximum 1–day precipitation	mm	Positive	Positive
RX5day	maximum 5–day precipitation	annual maximum consecutive 5–day precipitation	mm	Negative	Positive

*Source*: Results from New, M., Hewitson, B., Stephenson, D.B., Tsiga, A., Kruger, A., Manhique, A. *et al.*, 2006, ‘Evidence of trends in daily climate extremes over southern and west Africa’, *Journal of Geophysical Research – Atmospheres* 111, D14102. http://dx.doi.org/10.1029/2005JD006289

The regional trends for the abovementioned indices were obtained from New *et al.* (2006). Flood disaster events were identified using the global disaster databases. There are many disaster databases that catalogue flood disaster events using various criteria. These criteria resulted in underreporting of disasters in many developing countries such as Namibia, as the lack of efficient and effective disaster management communication systems – amongst others – are limiting the recording of small yet damaging events. The global databases that have information on Namibia include:

- EM-DAT (2015c)
- United Nations Office for the Coordination of Humanitarian Affairs (OCHA) – Reliefweb (2015
- International Flood Network (IFNET 2015)
- Dartmouth Flood Observatory (De Groeve 2010; Dartmouth Flood Observatory [DFO] 2015).

The DFO and EM-DAT databases have the most extensive records of flood events and their impacts, but underreporting, especially of relatively small and frequent floods, is a great obstacle to reliable validating risk estimates, in these databases and others (Adhikari *et al.* 2010; Smith 2013; Tschoegl *et al.* 2006).

Fourthly, the daily precipitation data of Grootfontein were used to derive the annual cumulative rainfall sum for each year and a least square trend line was calculated.

The regional averaged (spatial) climatologies for the region (Figure 2b, c) were derived from the CRU product using a latitude and longitude box (15°S – 20.1°S and 13.9° E – 19.4°E) and the standardised anomalies were calculated from the data that were also aggregated to an annual dataset whilst a moving average trend line (10–year) was also calculated (Figure 8; Washington & Preston 2006).

Published literature on reported flood events (precipitation based) were obtained and used to verify the reference flood events listed in Table 2.

These referenced flood events were compared to events derived from the extreme precipitation indices, literature, global disaster databases, the Grootfontein rainfall station's climatologies and regional averaged CRU data.

## Results

The flood disaster events mentioned in the media and elsewhere prompted questions about whether such events were becoming more frequent or not. An up to date archive is needed to inform and help in the reporting of unquantified disaster events, especially in an area such as the CRB. The CRB has a chronic shortage of long-term hydro-meteorological data that are also needed for rigorous frequency analyses. This can help with the reporting of whether an event can be classified as one of the worst flood disaster events or heaviest-ever rainfall events in the region (Grobler 2011; Paeth *et al.* 2011). Thus, this section used different methods to quantify the recorded flood events (Figure 4) and compare them to data derived from different sources.

### Grootfontein rainfall station

**FIGURE 6:**
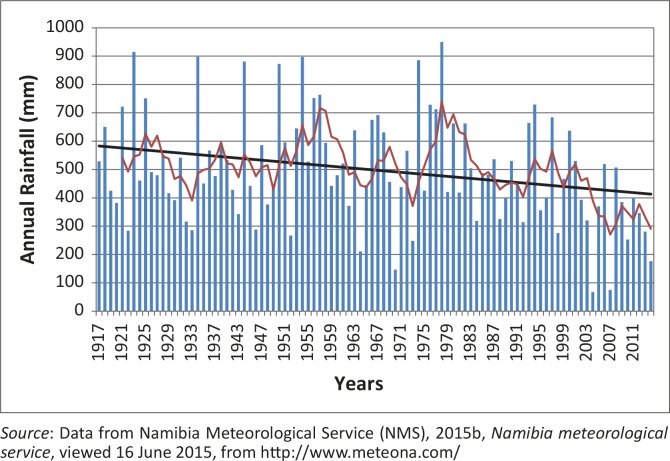
The annual rainfall of Grootfontein from 1917 to 2014 with a least square linear regression and 10–year moving average trend lines.

The annual precipitation of Grootfontein range from 1917 to 2014 (Figure 6) is presented. A least square linear regression line (straight) shows a declining trend for the Grootfontein annual rainfall time series whilst the 10–year moving average line emphasises the wet and dry spells experienced at the Grootfontein station. Data on flood hazard events are available for 73 years (approximately seven decades) from 1941 to 2013 (Figure 4 and Table 1; Mendelsohn *et al.* 2013). These seven decades will be used to derive wet and dry spells from the Grootfontein rainfall time series. The first decade (1940–1950) was one of the driest, using the long-term mean of 500 mm with only the years 1944 and 1950 recording exceptional rainfall (Rouault & Richard 2003; Washington & Preston 2006).

The second decade (1951–1960) started with dry conditions (1952 recorded only 267 mm), although it was one of the wettest periods in the entire time series. Other studies from southern Africa also reported drought conditions for the year 1952 (Rouault & Richard 2003; Washington & Preston 2006). For the decade the highest recorded rainfall was 897 mm (1954). This value was also confirmed by the studies mentioned above. Good rainfall conditions that could result in flooding continued from the second decade into the first half of the third decade (1961–1970) with 638 mm recorded for the year 1963 despite the remainder of the decade experiencing dry conditions. This pattern of dry conditions is again echoed by the studies mentioned earlier. The lowest rainfall during this decade was 147 mm (1970) and this low rainfall for this year was also highlighted by the earlier studies.

Dry conditions continued into the fourth decade (1971–1980) whilst wetter conditions occurred at the end of the decade. The highest annual accumulative rainfall of 950 mm (1978) was recorded whilst 886 mm (1974) was measured earlier. Rouault and Richard (2003) have reported floods for the years: 1972, 1974, 1975, 1976 and 1978 whilst Washington and Preston (2006) had also reported these years as wet periods.

The fifth decade (1981–1990) started with wet conditions and ended with dry conditions. The highest rainfall was 662 mm (1982) whilst the lowest within this decade was 319 mm (1984). Floods (1981, 1988 and 1989) and droughts (1982, 1983, 1984 and 1987) were reported by Rouault and Richard (2003) whilst Washington and Preston (2006) reported dry conditions between 1979 and 1987. According to DWA (2015) and Nilsson (2012), the Orange-Vaal basin at Upington (discharge station) recorded two of the highest monthly peak flows within the station's 72 year record.

The sixth decade (1991–2000) was relatively dry compared to the second and the fourth decades whilst the last decade (2001–2013) was the driest for the Grootfontein rainfall station. In the sixth decade, 729 mm (1994) was recorded whereas the lowest recorded annual accumulative rainfall figures of 68 mm (2004) and 75 mm (2007) were recorded in the last decade which was one of the driest decades (DWA 2015).

To summarise, using an annual accumulative total of 900 mm for the Grootfontein rainfall station, the wettest spells (highest seven totals), in decreasing order, can also be identified as: 1978 (950 mm), 1923 (915 mm), 1934 (898 mm), 1954 (897 mm), 1974 (885 mm), 1944 (881 mm) and 1950 (873 mm). Table 3 shows the descriptive statistics for the Grootfontein rainfall station (96–year time series).

**TABLE 3:** Descriptive statistics for the Grootfontein rainfall station derived from a 96–year time series: 1917–2014.

Statistical parameters	Values
Mean	498.1375
Standard Error	19.22688
Median	478.5
Mode	425
Standard Deviation	188.3842
Sample Variance	35488.61
Kurtosis	−0.00836
Skewness	0.325199
Range	882
Minimum	68.2
Maximum	950.2
Sum	47821.2
Count	96

### Extreme precipitation indices

Extreme precipitation indices were used to identity and validate flood events in this study. Identifying extreme precipitation events are important as these events are often associated with flood hazards and, ultimately, higher risk of the vector and epidemic diseases such as malaria and cholera (Anyamba *et al.* 2006). Floods can also be highly beneficial in drylands (especially in Africa) as the floodwaters infiltrate and recharge alluvial aquifers along ephemeral river pathways, extend water availability to dry seasons and drought years and also sustain rain-fed agriculture (Morin *et al.* 2009).

Regionally, studies have shown that the most extreme daily precipitation indices, over southern Africa, showed approximately identical proportions of increasing as well as decreasing trends for the subregion, even though a very small number of station trends are statistically significant for any index (New *et al.* 2006).

The southern African region has many diverse climatic zones which can therefore produce extensive trends in inter-annual and decadal-scale variability of rainfall, hence, secular trends would not be easily detected. New *et al.* (2006), reported that only three indices have statistically significant increasing trends in southern Africa: annual maximum 1–day precipitation (RX1day), average wet day precipitation (SDII) and maximum dry spell duration (CDD). They further reported that another three indices, the maximum 5–day precipitation (RX5day) and the total precipitation indices on extreme rainfall days (R95p and R99p) also show (non-significant) increasing regional trends. They also reported that there were decreasing trends (non-significant) for four indices: annual precipitation (PRCPTOT), heavy precipitation days (R10 mm and R20 mm) and CWD. They reported increases as well as decreases for the subregion, the former for average rainfall intensity and the amount of rainfall on extreme rainfall days and periods whilst the latter for the total rainfall as well as number of days with heavy rainfall (see Table 2 for the regional trends; New *et al.* 2006).

An increase of the CDD index over southern Africa was reported by New *et al*. (2006) and it is the only index that shows a consistent trend. As the indices used in this study are calculated on an annual basis, the CDD recorded dry spells (days) within a year and, therefore, highlighted the length of the dry season but could not effectively record the number of dry days that occurred in a particular rainy season.

Large parts of the region experience one long rainy season from October to April (Yuan *et al.* 2014) and the result from this index confirms the findings of other studies, notably, a shortening of the rainy season (Tadross, Hewitson & Usman 2005). The rural population of the region practice subsistence farming and a variability in seasonal characteristics such as the onset and cessation of rain, and dry spell frequency can be damaging to agriculture, and a short rainy season can be more disastrous. The last mentioned study, amongst others (Tadross, Hewitson & Usman 2003), investigated the inter-annual variability of the onset of the maize growing season on Zimbabwe and South Africa and found that the onset was starting later in the period between 1979 and 2001. These findings can enhance farmers’ understanding of the onset of the season and enable them to predict the planting of maize (a vital cereal in the region) as it requires consistent water during its germination phase and, thus, planting maize at the right time is vital resulting from many resource-constraints.

Table 2 summarises the trends for all ten extreme precipitation indices for the Grootfontein rainfall station and the regional (southern Africa) trend as positive (increasing) or negative (decreasing) trend, whilst Figure 7 shows the graphs of the ten indices calculated from daily rainfall data of the Grootfontein station. Because of the rigorous selection criteria of the Grootfontein station compared to other surrounding regional rainfall stations, data for years between 1968 and 1989 were not plotted, and all graphs have gaps for these years with the exception of the year 1975 (Figure 7).

**FIGURE 7:**
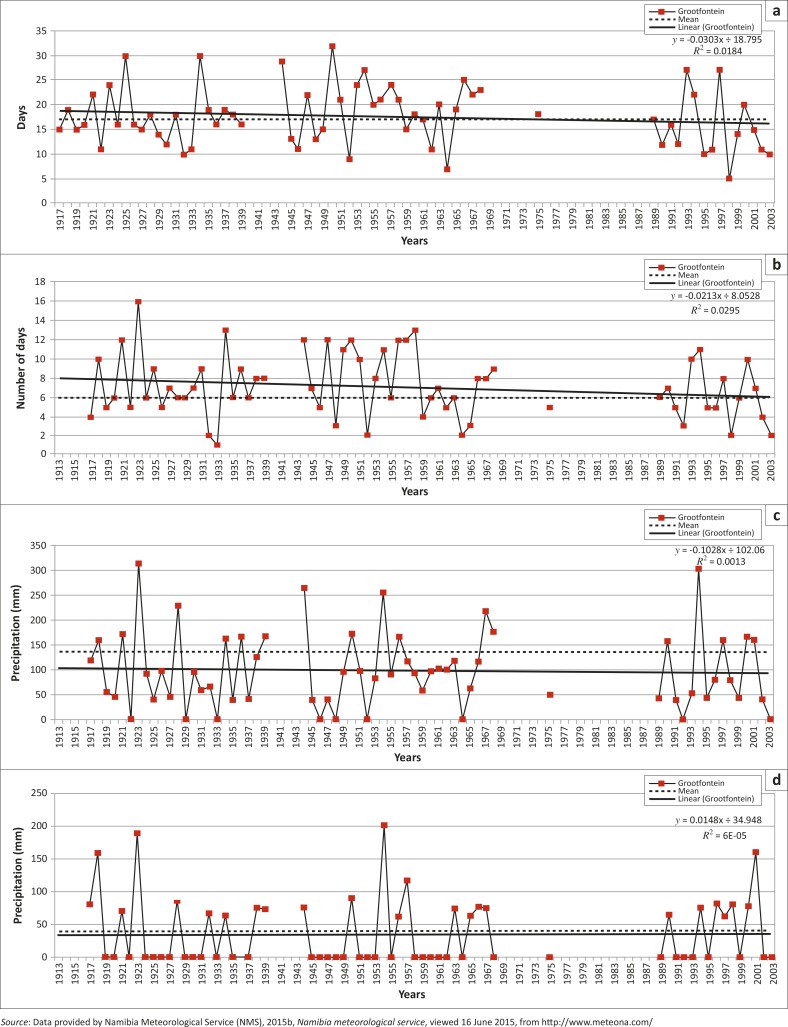
Ten climate change extreme precipitation indices for the Grootfontein rainfall station: (a) annual count of days when RR > 10 mm; (b) annual count of days when RR >= 20 mm; (c) annual total precipitation when RR > 95^th^ percentile of 1961–1990 daily rainfall; (d) annual total precipitation when RR > 99th percentile of 1961–1990 daily rainfall; (e) annual maximum 1–day precipitation; (f) annual maximum consecutive 5–day precipitation; (g) maximum number of consecutive wet days; (h) maximum number of consecutive dry days; (i) annual total precipitation from wet days and (j) average precipitation on wet days.

**FIGURE 7(Continues…):**
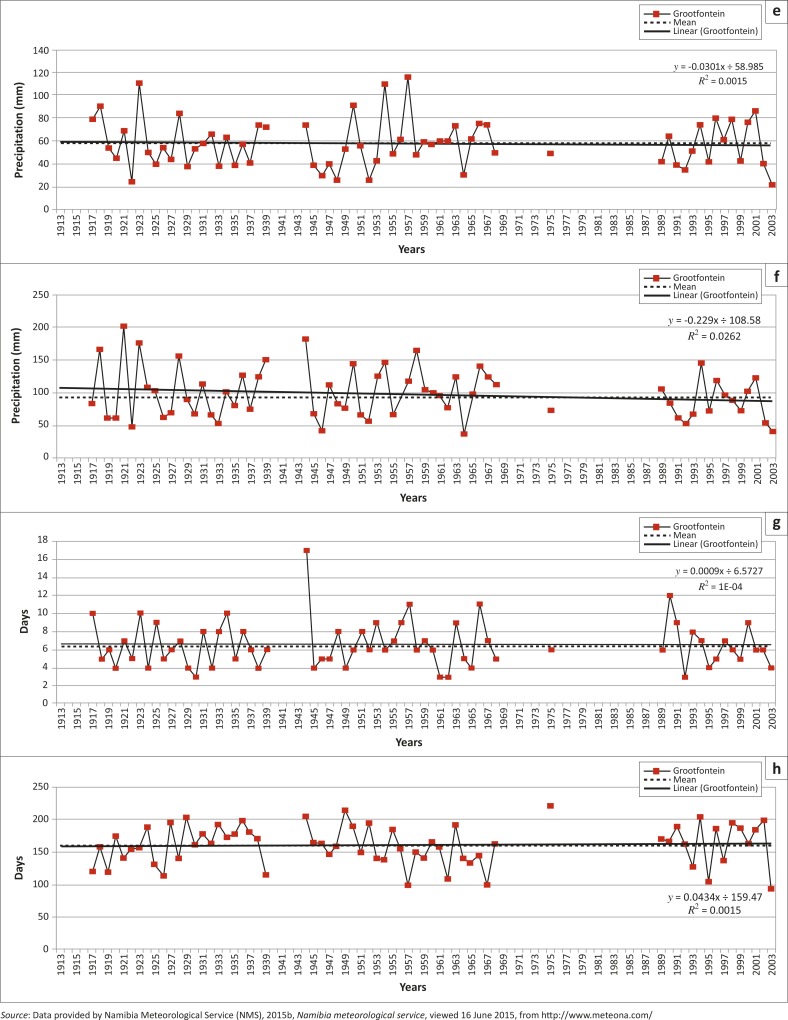
Ten climate change extreme precipitation indices for the Grootfontein rainfall station: (a) annual count of days when RR > 10 mm; (b) annual count of days when RR >= 20 mm; (c) annual total precipitation when RR > 95^th^ percentile of 1961–1990 daily rainfall; (d) annual total precipitation when RR > 99th percentile of 1961–1990 daily rainfall; (e) annual maximum 1–day precipitation; (f) annual maximum consecutive 5–day precipitation; (g) maximum number of consecutive wet days; (h) maximum number of consecutive dry days; (i) annual total precipitation from wet days and (j) average precipitation on wet days.

**FIGURE 7(Continues…):**
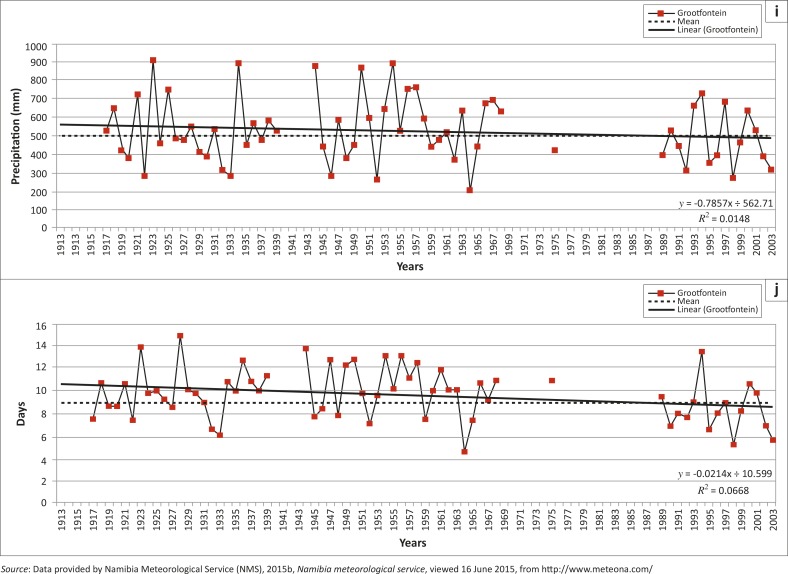
Ten climate change extreme precipitation indices for the Grootfontein rainfall station: (a) annual count of days when RR > 10 mm; (b) annual count of days when RR >= 20 mm; (c) annual total precipitation when RR > 95^th^ percentile of 1961–1990 daily rainfall; (d) annual total precipitation when RR > 99th percentile of 1961–1990 daily rainfall; (e) annual maximum 1–day precipitation; (f) annual maximum consecutive 5–day precipitation; (g) maximum number of consecutive wet days; (h) maximum number of consecutive dry days; (i) annual total precipitation from wet days and (j) average precipitation on wet days.

The increases for the extreme indices calculated for the Grootfontein station compared favourably to those reported by New *et al*. (2006). These were: RX1day and CDD, whilst non-significant increase was only for the R99p. Regionally, no significant decreases were found but a decrease was reported for the Grootfontein station (R10 mm index, Table 2).

### Regional Climate Research Unit data

**FIGURE 8:**
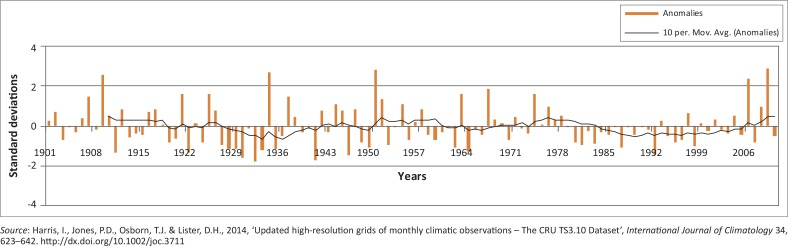
Standardised anomalies of the spatial (regional) averaged CRU data with a 10–year moving average trend line.

The inter-annual and decadal-scale variability of precipitation over the study area (spatially-averaged box around the Cuvelai basin) for CRU data are depicted in Figure 8. The 10–year moving average trendline illustrates the decadal-scale variability of precipitation whilst the anomalies are portrayed by bars using standard deviations. The first decade (1940–1950) was very variable with six negative anomalies and five positive anomalies. The highest negative anomaly occurred in 1941 (-1.7) whilst the highest positive anomaly was in 1950 (2.8). The trend line for the second decade (1951–1960) shows positive anomalies representing above normal rainfall conditions, although this decade had four positive standard anomalous years and five negative anomalous years. The highest positive anomaly was in 1951 (1.35) whilst the lowest negative anomaly was in the following year, 1952 (-0.93). The trend line for the third decade (1961–1970) shows that it was a below average decade with dry conditions, which are reflected with the four positive and the five negative anomalies with the highest positive being in 1967 (1.83) and lowest negative in 1970 (-0.67). These 2 years correspond with wet periods (floods) in South Africa and dry conditions (droughts) respectively (DWA 2015; Nilsson 2012; Rouault & Richard 2003; Washington & Preston 2006). As in the second decade, the trend line for the fourth decade (1971–1980) shows above normal rainfall conditions reaching a peak in 1974, flood hazard conditions characterised this year, as confirmed by the aforementioned studies. The fourth decade also had six positive and four negative anomalous years with the highest positive anomaly being in 1974 (1.6) whilst the lowest negative anomaly was in 1980 (-0.81). Studies (mentioned earlier) in South Africa reported flood events for most of these six positive anomalies: 1974, 1975, 1976, 1977 and 1978. The next two decades (1981–1990 and 1991–2001) experienced exceptionally dry conditions peaking in 1987 (-1.06) and in 1992 (-1.43), respectively, for these two decades. These 2 years had major drought conditions in South Africa as well as in the region (DWA 2015; Nilsson 2012; Rouault & Richard 2003; Washington & Preston 2006). In these two decades, the only two positive anomalous years were 1993 (0.28) and 1997 (0.62). The dry conditions prevailed during the first part of the seventh decade (2001–2010), according to the trend line (Figure 8) and five positive as well as five negative anomalous years are shown in Figure 8 with the highest positive anomalous year being 2009 (2.88) and the lowest negative anomaly year falling in 2007 (-0.80).

## Discussion

Table 4 summarises the different sources (hydro-meteorological data, global disaster data bases, extreme precipitation indices, and published disaster events in literature) used in this article to identify flood events for the last seven decades for which data for flood events (as referenced) are available. The referenced flood events that were used by this article were shown in Figure 4 and Table 1.

**TABLE 4:** Flood disaster events archive compiled from hydro-meteorological data, global disaster data bases, extreme precipitation indices and disaster events published in literature.

Decades	Table 1	Hydrologic modelled or annual observed flood peaks	Published events (Precipitation based)	Global disaster archives	Climatology	Extreme precipitation indices
1940 – 1950	8 flood events: 1949H, 1943M, 1946M, 1941L, 1942L, 1944L, 1947L, *&* **1950**L	BAR Namibia (2012): **1944**H, **1950**H, **1947**M, 1940L, 1942L, 1945L, 1949L	Engert (1997)*:* Wet spells: **1950**, **1944**, 1940, 1942, 1948 *&* **1947**	-	Grootfontein: 1944, 1947 *&* **1950**	R20 mm: **1944**, 1945, **1947**, 1949 *&* **1950**
						R95p: **1944** *&* **1950**
						R99p: **1944** *&* **1950**
		Van Langenhove (2011)*:* Rundu – **1947**, 1948 *&* **1950**	DWA (1990): ‘Wet’ year – **1950**		CRU: 1942, 1944, 1945, 1947 *&* **1950**	
						PRCPTOT: **1944**, **1947** *&* **1950**
		DWA (2015) *&* Nilsson (2012)*: 1944 & 1948*	Rouault and Richard (2003)*:* Droughts: 1945, 1947 *&* 1949 and Floods: 1948			RX1day: **1944** *&* **1950**
						RX5day: **1944**, **1947** *&* **1950**
						SDII: **1944**, **1947**, 1949 *&* **1950**
						CWD: **1944** *&* 1948
						CDD: **1944**, 1945, 1946, 1949 *&* **1950**
1951 – 1960	4 flood events: 1953H, **1956**H, **1955**M, 1958L	BAR Namibia (2012): 1951L *&* 1954L	Engert (1997)*:* Wet spells: 1954, **1956**, 1958, 1955	-	Grootfontein: 1951, 1953, 1954, **1955**, **1956**, 1957 *&* 1958	R20 mm: 1951, 1953, **1956**, 1957, *&* 1958
						R95p: 1954 *&* **1956**
		Van Langenhove (2011)*: Rund*u – 1951 *&* 1954	Jury (2010)*:* ‘Wet’ season – 1954			R99p: 1954, **1956** *&* 1957
						PRCPTOT: 1951, 1953, 1954, **1955**, **1956**, 1957, 1958,
						RX1day: 1954, **1956** *&* 1957
		DWA (2015) *&* Nilsson (2012)*:* **1955** and 1957	Rouault and Richard (2003)*:* Droughts: 1952 and Floods: **1955**		CRU: 1951, 1954, **1956**, *&* 1957	
						RX5day: 1953, 1954, **1956**, 1957, 1958, 1959 *&* 1960
						SDII: 1950, 1951, 1953, 1954, **1955**, **1956**, 1957, 1958 *&* 1960
						CWD: 1951, 1953, **1955**, **1956**, 1975 *&* 1959
						CDD: 1952, 1955 *&* 1960
1961 – 1970	No records: 1960 –1968	BAR Namibia (2012): **1963L** *&* 1968L	Engert (1997)*:* Wet spells: 1968, **1963**, 1966, **1967** *&* 1969	-	Grootfontein: 1961, **1963**, 1966, **1967** *&* 1968	R20 mm: 1961, 1966 *&* **1967**
	1 flood event: 1970H					R95p: **1967** *&* 1968
						R99p: **1963**, 1965, 1966 *&* **1967**
		Van Langenhove (2011)*:* Rundu – 1961, 1962, **1963**, 1965, 196, **1967**, 1968, 1969 *&* 1970	Jury (2010)*:* ‘Wet’ seasons –1962, **1963**, 1968 *&* 1969			
						PRCPTOT: 1961, **1963**, 1966, **1967** *&* 1968
						RX1day: 1961, 1962, **1963**, 1965, 1966 *&* **1967**
						RX5day: 1961, **1963**, 1965, 1966, 1968 *&* 1968
					CRU: **1963**, **1967** *&* 1968	
		DWA (2015) *&* Nilsson (2012)*:* 1966 and **1967**	Rouault and Richard (2003)*: Droughts: 1964, 1965, 1966, 1968 & 1970 and* Floods: 1961, **1963** *&* **1967**			SDII: 1961, 1962, **1963**, 1966, **1967** *&* 1968
						CWD: **1963**, 1966 *&* **1967**
						CDD: 1963 *&* 1968
1971 – 1980	4 flood events: **1976**H, 1977H, **1975**M *&* 1979M	BAR Namibia (2012): 1971M, 1974M, **1976**M, **1978M** *&* 1979L	Engert (1997)*:* Wet spells: **1976**, 1974, **1978**, 1979, 1971, 1972 *&* **1975**	-	Grootfontein: 1972, 1974, **1976**, 1977, **1978**, *&* 1980	SDII: **1975**
						CDD: 1975
			Jury (2010)*:* ‘Wet’ season – 1979			
		Van Langenhove (2011)*:Rundu* – **1975**, **1976**, 1977 *&* 1979				
			Jury *&* Majodina (1997)*:* Rainfall exceedance > 70mm – 1974 *&* **1978**			
					CRU: 1971, 1974, **1975**, **1976**, 1977 *&* **1978**	
		DWA (2015) *&* Nilsson (2012)*:* 1974 *&* **1976**				
			Rouault and Richard (2003)*:* Droughts: 1979 and Floods: 1972, 1974, **1975**, **1976** *&* 1978			
1981 – 1990	6 flood events: 1983M, 1985M, 1987M, 1982L, **1984**L *&* 1990L	Van Langenhove (2011)*:* Rundu – 1981, **1984** *&* 1986	Engert (1997)*:* Wet spells: 1989, 1982, 1986 *&* **1984**	-	Grootfontein: 1982, 1987 *&* 1990	R20mm: 1990
						R95p: 1990
						R99p: 1990, 1996, 1997 *&* 1998
			Jury (2010)*:* ‘Wet’ seasons – **1984**			
						PRCPTOT: 1990
						RX1day: 1990
						RX5day: 1989, 1990
						SDII: 1989
		DWA (2015) *&* Nilsson (2012)*:* 1988	Jury *&* Majodina (1997)*:* Rainfall exceedance > 70mm – 1988			
						CWD: 1990
						CDD: 1989 *&* 1990
			Rouault and Richard (2003)*:* Droughts: 1982, 1983, 1984 *&* 1987 and Floods: 1981, 1988 *&* 1989			
1991–2000	9 flood events: 1995H, 1991M, 1994M, 1997M, 1999M, **2000**M, 1993L, 1996L *&* 1998L	BAR Namibia (2012): **2000**L	Jury (2010)*:* Flood event (3–day totals) – **2000**	-	Grootfontein: 1993, 1994, 1997 *&* 2000	R20 mm: 1993, 1994, 1997, **2000**
						R95p: 1994, 1997, *&* **2000**
						R99p: **2000**
						PRCPTOT: 1993, 1994, 1997 *&* **2000**
		Van Langenhove (2008)*:* 1995			
						RX1day: 1994, 1996, 1997, 1998 *&* **2000**
			Rouault and Richard (2003)*:* Droughts: 1992, 1993 *&* 1998 and Floods: 1996 *&* 1997			RX5day: 1994, 1996, 1997 *&* **2000**
						SDII: 1994 *&* **2000**
					CRU: 1991, 1997*&* 1999	
		Van Langenhove (2011)*:* Rundu – 1992 *&* 1999				
						CWD: 1991, 1993, 1994, 1997 *&* **2000**
						CDD: 1991, 1992, 1994, 1996, 1998, 1999 *&* **2000**
2001 – 2013	9 flood events: 2004H**, 2008**H, **2009**H, **2011**H, **2001**M, 2005M, 2006M, **2010**M *&* 2012M	BAR Namibia (2012): **2011**M, **2008**L, **2009**L *&* **2010**L	Jury (2010)*:* ‘Wet’ season – 2004 *&* Flood events (3–day totals) – 2003, 2002 *&* **2001**	EM-DAT (2015c): 4 flood events – 2004, **2008**, **2009**, **2010** *&* **2011**	Grootfontein: **2001**, 2006 *&* **2008**	R20 mm: **2001**
						R95p: **2001**
						R99p: **2001**
		Van Langenhove (2008)*:* 2008				
			NMS (2015a): Ondangwa – **2011**, **2009**, 2006, **2008**, **2010** *&* 2012			PRCPTOT: **2001**
		Mufeti and Katjizeu (2013) *& NHS (2015):* **2008**, **2009**, **2010** *&* **2011**				RX1day: **2001**
					CRU: **2001**, 2004, 2006, **2008** *&* **2009**	RX5day: **2001**
						SDII: **2001**
		Van Langenhove (2011)*:* Rundu – **2001**, 2004, 2005, 2007, **2009**, **2010** *&* **2011**				
						CWD: -
						CDD: **2001** *&* 2002
		DWA (2015) *&* Nilsson (2012)*:* **2011**				

Note: Common years amongst the different sources are in bold (for example 1944, 1947 and 1950).

H, Major flood disaster (high flows); M, Medium floods (medium flows); L, negligible flow (Small floods); BAR: H = 160–300m^3^/s; M = 301–500m^3^/s; L = 501–700m^3^/s

For the first decade (1940–1950) eight flood disaster events were reported and three of these events were reported by BAR Namibia (2012), Engert (1997), climatologies derived from CRU, Grootfontein station rainfall data as well as from a few extreme precipitation indices. All the major sources, listed in Table 4, reported the years 1944, 1947 and 1950 as the most likely years for extreme precipitation and hence flooding. Figure 8 shows that this decade had experienced a dry spell except for the year 1944 when the long-term (96 years) mean of 500 mm was used. Figure 8 also shows the standardised anomalies derived from spatially and time-averaged CRU (monthly) data and confirmed that this decade had minimal rainfall. This decade was also preceded with a very dry period between 1927 and 1942. Other studies such as Washington and Preston (2006) confirmed wet years for 1944 and 1948 whilst the Orange-Vaal basin at the Upington discharge gauge also recorded high peak flows for these 2 years (DWA 2015; Nilsson 2012).

The second decade (1951–1960) had only four referenced events and only one flood disaster event (1956), which was commonly occurring amongst the different sources used to identify them. Figures 7 and 8 confirm, using 10–year moving averages, that this decade was one of the wettest periods for Grootfontein and the region. This period had the most possible flood disaster events, as illustrated by the extreme precipitation indices. Other studies such as Washington and Preston (2006) confirmed these possible wet periods: 1953, 1954, 1954, 1955, 1956 and 1957, whilst the Orange-Vaal basin at the Upington discharge gauge also recorded high peak flows for 2 of these years: 1955 and 1957 (DWA 2015; Nilsson 2012).

The third decade (1961–1970) lacks sufficient observational data for the period 1960–1968 (Table 4) but one flood event (1970) was reported during that period as a referenced flood event (Table 1). However, 1963 was the most common year in which a flood was reported by the different sources consulted (Figure 8). The first half of the decade is recorded as very dry whilst the second half had reasonable wet spells. Overall, Figure 8 (10–year moving average) indicates a dry decade regionally whilst 1967 was a very wet year. Other studies such as Washington and Preston (2006) confirmed these possible dry periods: 1961, 1962, 1964, 1965, 1966, 1968 and 1970 as well as wet periods: 1963, 1967 and 1969 whilst the Orange-Vaal basin at the Upington discharge gauge recorded high peak flows for 2 years: 1966 and 1967 (DWA 2015; Nilsson 2012).

The fourth decade (1971–1980) had the fewest recorded flood events from all sources, only one index (SDII) reported an extreme event for 1975. Generally, 1975 was reported by most of the sources as a year during which flood events had occurred. This decade also began with dry spells and ends with wet spells that included extreme precipitation for 1974 and 1978 for the Grootfontein station. Regionally, it was a wet year with only 1973 appearing to be a dry year. The extreme precipitation indices failed to record this, as these years were not plotted, resulting from the rigorous rainfall selection criteria. Other studies such as Washington and Preston (2006) reported three dry periods: 1973, 1979 and 1980 as well as wet periods: 1971, 1972, 1974, 1975, 1976, 1977, 1978 and 1979, whilst the Orange-Vaal basin at the Upington discharge gauge recorded high peak flows for 2 years: 1974 and 1976 (DWA 2015; Nilsson 2012).

The fifth decade (1981–1990) started as a wet year (Figures 7 and 8) but ended with dry conditions. Table 4 shows that 1984 was the most commonly reported year for flood events even though the extreme precipitation events failed to report it, resulting from reasons given earlier for the period 1968–1989. This decade started with wet conditions and ended with the worst drought (1991–1992) reported in southern Africa (Benson & Clay 1998). Figure 7 illustrates the period of the drought conditions from 1983–2004 with good rainfall only in the years 1993, 1997, 1999, 2001 and 2004. Other studies such as Washington and Preston (2006) reported a dry period from 1981 until 1987 and wet periods only for: 1988 and 1989, whilst the Orange-Vaal basin at the Upington discharge gauge recorded its highest peak flow: 1988 (DWA 2015; Nilsson 2012).

The sixth decade covers the period 1991–2000 and includes the driest periods in southern Africa. The CDD index reported 7 years as very dry periods whilst the year 2000 is widely reported (Table 4) as a year for a flood event. Figure 7 confirms the five good rainfall years for the Grootfontein station, even though Washington and Preston (2006) only reported three wet periods: 1991, 1997 and 1998, whilst the rest of the decade was dry. The Orange-Vaal basin in South Africa also experienced severe drought conditions, hence the Upington discharge gauge did not record any high flow peaks for this decade (DWA 2015; Nilsson 2012).

For the last decade (2001–2013) the highest number of reported flood events was nine (Table 4). The years 2008, 2009, 2010 and 2011 were widely reported (Figure 3 and Table 4) as years when flood disaster events occurred. Regionally, this period started with dry conditions and recorded the most consecutive flood disaster events in Namibia, causing severe damage. These consecutive flood events occurred during the latter part of this decade. In general, the annual precipitation for the Grootfontein station shows a significant decreasing trend (Figure 6), whilst Figure 7 confirmed the same dry years whilst these wet spells (consecutive flood events) are also highlighted. The Upington discharge gauge recorded a high flow peak for 2011 (DWA 2015; Nilsson 2012).

One of the main objectives of this study is to identify drought and flood events during periods where no observational hydro-meteorological data are available (Figure 4 & Table 1). The most incomplete observational records are reported for the third decade (1961–1970), of 13 years (Table 1). These 13 years are within the climate normal period (1961–1990) used by climatologists to compare climatological trends to that of the past, or what is considered ‘normal’, hence when the same years within this period are absent, indices will not be calculated, as in this article (1968–1989). Using the methods followed in this article, hydro-meteorological events were assigned to 10 of the 13 years. A few years (1965, 1966 & 1968) yielded contradicting results (events), for example, these 3 years were reported as droughts in South Africa, whilst several sources reported them as flood hazard years in Namibia. This article assumed that the dominant rain-producing systems in the region have a large enough spatial extent to be well represented in the different data sources used for the subregion (Tadross *et al.* 2003, 2005). Future work should, therefore, investigate these and other differences (hydro-meteorological events) that might have resulted from local topography or other uncertainties. Additionally, this article did not calculate the magnitude of these hazardous events, hence rigorous frequency analysis should be performed on both available modelled and observed flow and stage data as well on satellite-based rainfall estimates and land-based rainfall gauge data. This would be a challenging task in an ungauged and under gauged basin such as the Cuvelai.

Lastly, as a result of increasing changes in the climate, land-use and extreme precipitation, disaster risk reduction and flood risk management strategies are essential in adapting to the envisaged increasing flood risk. This necessitates an accurate inventory of hydro-meteorological events, which can also be updated to provide a quantified spatial and temporal distribution of floods, including magnitude, frequency, and duration. The major limitation of this article is that it does not include methods that can help with the identification of historical records of catastrophic floods, such as records of physical signs of water levels on old buildings, memories of old citizens, historical documents, news reports and archive reports from meteorological and hydrological national services as well as detail discharge and rainfall frequency analysis resulting from data scarcity.

## Conclusion

The present contribution produced an up to date archive of flood events (years) by validating and supplementing referenced flood events, and also comparing them against indices of extreme precipitation (years) derived from daily climate data as well as data derived from other sources for northern Namibia.

Generally, for the extreme precipitation indices, no significant regional trends were identified, which does not come as a surprise for a continent where different factors affect regional rainfall, and where there are few consistent and statistically significant trends in extreme precipitation indices. Also, for the statistical regional trends, similar to other studies: an increase in average dry spell length was found for other indices such as the average rainfall intensity and annual 1–day maximum rainfall indices, whilst decreasing trends were found for the Grootfontein station. Furthermore, for the Grootfontein station, there is an indication of decreasing trends, for both total annual precipitation as well as for the total number of wet days annual precipitation.

The study appended the hydro-meteorological records, for the developed archive, for the ‘no records’ period of 1960–1968 by adding the years 1963 and 1967 and possibly 1968 as flood prone years. It also identified years when floods would have not been possible and, hence, these years require in-depth hydrologic and hydraulic modelling to confirm the occurrences of flood hazard events.

These findings highlighted the lack of and difficulty with obtaining observed hydro-meteorological data and performing analyses on them in Africa, where a large rural population entirely relies on precipitation for its water supply. This also, ultimately, determines strategies of food production and also the mobility of migrant groups.

The results from this article will provide an improved understanding of past extreme precipitation events that are required for scientists, practitioners, policymakers and civil society to better compare and refer to past and present flood and drought hazard events.

This article updated the inventory of past flood events by using different sources such as: hydro-meteorological data, global disaster databases, climate change extreme precipitation indices and disaster events published in literature.
